# Sudden Loss of Consciousness in the Operating Room: A Case of Concomitant Stroke in a Patient With Embolic Ischemia of the Limbs

**DOI:** 10.7759/cureus.95775

**Published:** 2025-10-30

**Authors:** Ricardo Portela e Silva, André Postiga, Mona-Lisa Coutinho, Miguel Paiva, Nídia Gonçalves

**Affiliations:** 1 Anesthesiology, Unidade Local de Saúde de Coimbra, Coimbra, PRT

**Keywords:** acute limb ischemia, atrial fibrillation, basilar artery occlusion, coma, thromboembolic events, transient ischemic attack, vertebrobasilar stroke

## Abstract

Atrial fibrillation (AF) is associated with substantial mortality and morbidity, mainly due to thromboembolic complications such as stroke, ischemic heart disease, and arterial thromboembolic occlusion. Occasionally, these events can occur simultaneously, even in patients under long-term anticoagulation. We present a case of a 79-year-old woman with a history of AF and anticoagulation, proposed for an emergent surgical thromboembolectomy due to acute embolic ischemia of the right lower limb. Upon arrival at the operating room (OR), she was conscious and fully aware, answering the anesthesiologist's questions when, suddenly, she experienced a loss of consciousness with apnea and a Glasgow Coma Scale (GCS) of 3. A head and neck computed tomography angiography (CTA) showed no acute lesions and no evidence of occlusion of the main intracranial and neck vessels. During transport to the radiology department, the patient began to slowly regain consciousness, and after the examination, she was, again, conscious and cooperative, without evident neurological deficits. After discussion with the stroke unit team, the most probable diagnosis was a vertebrobasilar territory transient ischemic attack (TIA) with quick spontaneous reperfusion. Vertebrobasilar territory ischemic events have a wide variety of presentations, with some patients presenting with coma. A concomitant ischemic stroke in a patient with an arterial thromboembolic occlusion is a rarely reported event, with potential for serious morbidity and mortality if not promptly managed. This case highlights the importance of being alert for these concomitant events, at any time, in the care of patients with other thromboembolic complications. Clinicians should maintain a high level of suspicion because the swift identification and treatment of stroke are essential to improve outcomes.

## Introduction

Atrial fibrillation (AF) is one of the most commonly encountered heart conditions, with a global prevalence estimated at 50 million in 2020 and expected to double in the next few decades [1,2]. AF is associated with substantial mortality and morbidity, mainly due to thromboembolic complications such as stroke, ischemic heart disease, and arterial thromboembolic occlusion. Occasionally, these thromboembolic complications can occur simultaneously, such as in the case presented in this article. Being a relatively rare occurrence, its reporting and dissemination are of particular importance.

The pathogenic mechanisms of thrombus formation in AF are incompletely understood, but the contribution of the processes described in Virchow's triad is well documented, namely, abnormal atrial wall structural changes, abnormal blood flow, and platelet and coagulation abnormalities [3,4]. Long-term anticoagulation is a routine therapeutic tool for the prevention of these events in patients with AF and high risk of thromboembolic events.

Ischemic stroke is a potentially fatal condition capable of causing devastating disabilities if not promptly managed and treated. Patients with AF have a two- to threefold increase in the relative risk (RR) of ischemic stroke (RR, 2.3; 95% CI, 1.84-2.94) [5]. Although anticoagulation significantly reduces these events, there is still a residual risk. One-third of patients with AF presenting with an ischemic stroke are already on anticoagulation [6,7]. Arterial thromboembolic occlusions of the limbs also represent a major part of thromboembolic complications in AF, with an estimated incidence of 0.4% annually [8].

A concomitant stroke in a patient with acute limb ischemia is a rarely reported but potential event, especially in patients with a known embolic source, such as AF. Stroke recognition in this setting can be challenging due to several factors: (a) patient characteristics, typically elderly, frail individuals with multiple comorbidities; (b) the wide spectrum of clinical presentations, ranging from asymptomatic cases to sudden coma-like states, as observed in our patient; and (c) contextual circumstances; in this case, the patient was already in the operating room (OR), about to undergo anesthesia for a femoral thromboembolectomy.

Clinicians should, at all times, maintain a high level of suspicion for these events due to the time-sensitive treatment options and the potential for serious sequelae of ischemic strokes.

## Case presentation

A 79-year-old woman was proposed for an emergent surgical thromboembolectomy due to acute embolic ischemia of the right lower limb. She entered the emergency department with pain and paresthesias in the right leg, paralysis of the right foot, and the absence of a popliteal pulse. Five thousand international units of intravenous unfractionated heparin were administered, and surgery was discussed with the patient, who consented to the procedure. She had a history of atrial fibrillation (anticoagulated with oral edoxaban 30 mg daily, with good compliance), hypertension, dyslipidemia, chronic obstructive pulmonary disease, and an ischemic stroke, 10 years prior, with no residual deficits. Laboratory values showed only a stage 1 (Kidney Disease: Improving Global Outcomes {KDIGO} criteria) acute kidney injury with increased serum creatinine.

Upon arrival at the OR, she was conscious and oriented in space, time, and person, answering the anesthesiologist's questions. Suddenly, before any drugs were administered, she experienced a sudden loss of consciousness with apnea. Assisted ventilation with a facial mask was quickly initiated, and an airway, breathing, circulation, disability, and exposure (ABCDE) assessment was performed. Her airway was unobstructed; there was no ventilatory drive and no resistance to manual insufflations. Her peripheral saturation was ≥99% with a fraction of inspired oxygen around 90%-100%. She was hemodynamically stable, with blood pressure of 127/71 mmHg and AF with a heart rate around 70 beats per minute (bpm). Neurologically, her Glasgow Coma Scale (GCS) was 3 (E1V1M1). Her pupils were isochoric in the midline, nonreactive to light, and she had no corneal reflex. She was normoglycemic and normothermic. With these findings, the team decided on endotracheal intubation (ETI) and controlled ventilation, and an emergent neurology consult was requested for the OR. No drugs, neither hypnotics nor neuromuscular blocking agents, were used in the ETI. The patient exhibited no movements or hemodynamic variations in response to the ETI, accomplished via direct laryngoscopy. Given the sudden onset of symptoms and the neurological findings, a neurological cause for the altered state of consciousness was considered the most likely. The neurologist arrived within the first 10 minutes after symptom onset, confirmed our neurological findings, and recommended a head and neck computed tomography angiography (CTA).

**Figure 1:**
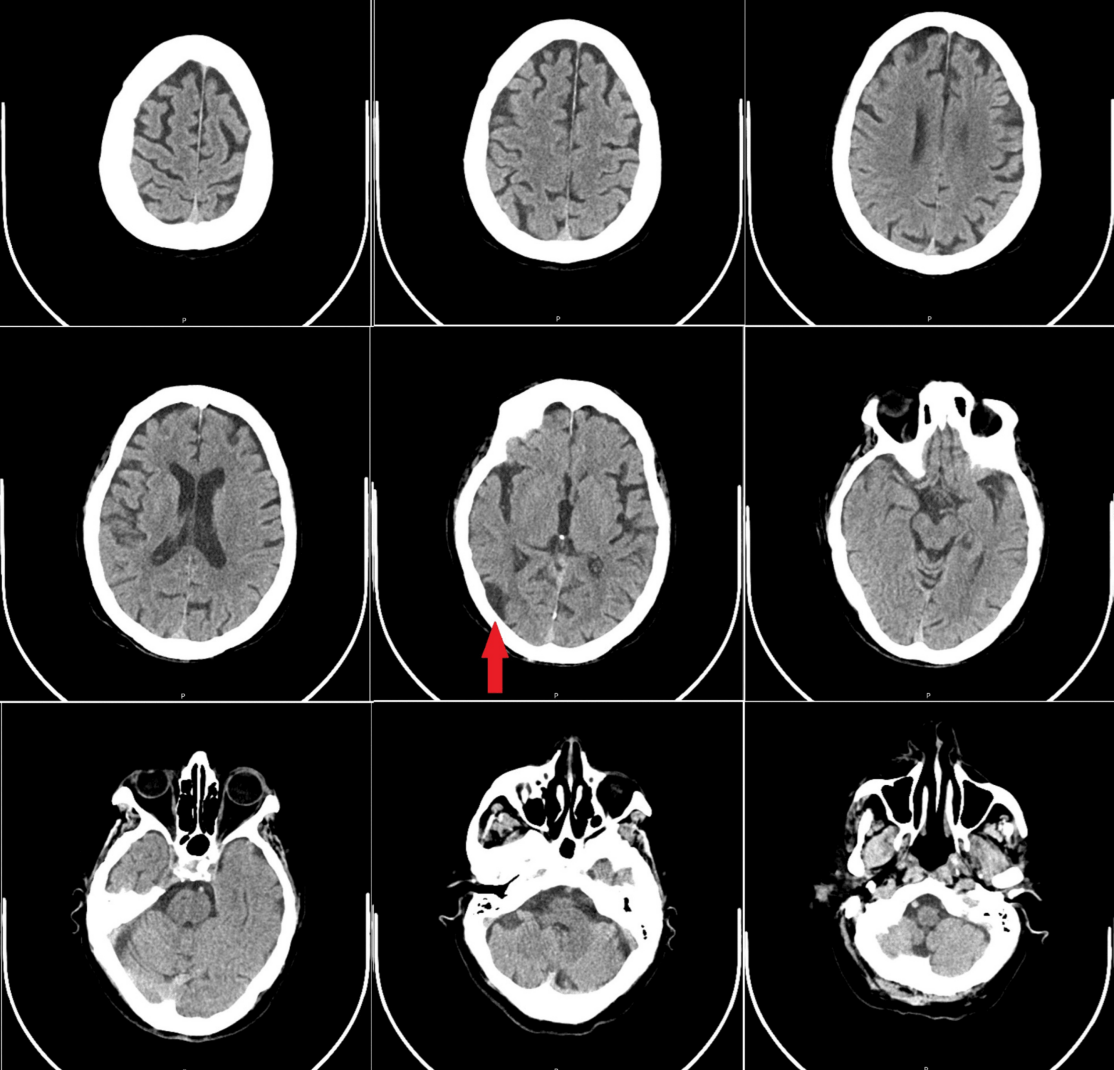
Computed tomography axial images showing no acute lesions; old right occipito-temporal infarction (red arrow)

During transport to the radiology department, approximately 15-20 minutes after the event, the patient began to slowly regain consciousness with some limb movement and a GCS of 7t (E2VtM5). The head and neck computed tomography (CT) and CTA showed only an old right occipito-temporal infarction but no acute lesions, no cortical hypodensity or sulcal effacement, and no evidence of occlusion or dissection of the main intracranial and neck vessels (Figure 1).

Shortly after the examination, the patient was, again, conscious and cooperative, without evident motor deficits and with protective airway reflexes and maintained spontaneous ventilation. She was transferred to the postanesthesia care unit (PACU), where she was extubated without complications within the first hour after the event. She had no neurological deficits and had amnesia for the event. Lower limb symptoms were improving with the initial anticoagulation, and she had regained motricity of the foot. Surgery was postponed, and she was transferred to the stroke unit for monitoring.

There was no recurrence of symptoms in her 24-hour stay in the stroke unit, where she kept anticoagulation with subcutaneous enoxaparin 1 mg/kg twice per day (bid). After discussion with the neurology and the stroke unit team, the most probable diagnosis was a vertebrobasilar territory transient ischemic attack (TIA) with quick spontaneous reperfusion.

## Discussion

The sudden onset of symptoms, the presence of thromboembolic risk, and the neurological findings are most consistent with a cerebrovascular cause of coma, although further investigations, such as magnetic resonance imaging, would be required to confirm the diagnosis. Considering other etiologies, no medications were administered, and the patient remained hemodynamically stable, making drug-induced or cardiac causes less likely. The patient had no history of epilepsy or seizures. The abrupt onset of coma without preceding symptoms is inconsistent with metabolic or infectious etiologies, and there were no grounds to suspect toxic ingestions.

Vertebrobasilar territory ischemic events, presumably the diagnosis of our patient, have a wide variety of presentations. Basilar artery occlusion (BAO) accounts for only about 1% of all ischemic strokes, but up to 30%-60% of patients might present with coma [9]. This results typically from ischemia of the reticular activating system, vital in sustaining consciousness. Embolic occlusion with rapid spontaneous recanalization can cause the transient loss of consciousness without other localizing deficits [10].

Stroke, in the setting of AF, is more likely to be fatal or associated with longer hospitalization, greater disability, and higher rates of recurrence [8]. In addition, most patients with BAO can have poor functional outcomes [11]. Fortunately, this was not the case.

A concomitant ischemic stroke in a patient with an arterial thromboembolic occlusion is a rarely reported event, with potential for serious morbidity and mortality if not promptly managed [12]. AF is associated with complex mechanisms of thromboembolism formation that go beyond the simple structural and mechanical abnormalities of the left atria [3,4]. While anticoagulation reduces the risk of thromboembolic complications, it does not abolish them [6,7].

This case highlights the importance of being alert for these events, at any time, in the care of patients with other thromboembolic complications, especially in the case of active AF, even with anticoagulation. Had the TIA occurred only moments later, potentially under general anesthesia during surgery, its recognition would have been much more challenging. The key factor to the success of stroke therapies, both intravenous fibrinolysis and mechanical thrombectomy, is early treatment, hence the importance of maintaining a high level of suspicion for its swift identification.

## Conclusions

While anticoagulation reduces the risk of thromboembolic complications in AF, it does not abolish them. A concomitant ischemic stroke in a patient with an arterial thromboembolic occlusion is a rarely reported but clinically significant event that can complicate diagnosis and delay time-sensitive interventions. Ischemic stroke is a potentially fatal condition, capable of causing significant disabilities if not promptly managed and treated. Clinicians, in the care of a patient with other thromboembolic complications, should maintain a high level of suspicion for these concomitant events, even under anticoagulation. The swift identification and treatment of stroke are essential to improve outcomes.
